# Non-obtrusive monitoring of obstructive sleep apnea syndrome based on ballistocardiography: a preliminary study

**DOI:** 10.3389/fnins.2025.1549783

**Published:** 2025-03-20

**Authors:** Biyong Zhang, Zheng Peng, Chunjiao Dong, Jun Hu, Xi Long, Tan Lyu, Peilin Lu

**Affiliations:** ^1^Eindhoven University of Technology, Eindhoven, Netherlands; ^2^Bobo Technology Ltd., Zhejiang, China; ^3^Department of Medical Imaging, Hebei Medical University, Shijiazhuang, China; ^4^Department of Electrocardiography, Sir Run Run Shaw Hospital, Zhejiang University School of Medicine, Hangzhou, China; ^5^Department of Neurology, Sir Run Run Shaw Hospital, Zhejiang University School of Medicine, Hangzhou, China

**Keywords:** obstructive sleep apnea syndrome, apnea-hypopnea index, ballistocardiography, non-obtrusive monitoring, machine learning

## Abstract

**Introduction:**

Obstructive sleep apnea syndrome (OSAS) degrades sleep quality and is associated with serious health conditions. Instead of the gold-standard polysomnography requiring complex equipment and expertise, a non-obtrusive device such as ballistocardiography (BCG) is more suitable for home-based continuous monitoring of OSAS, which has shown promising results in previous studies. However, often due to the limited storage and computing resource, also preferred by venders, the high computational cost in many existing BCG-based methods would practically limit the deployment for home monitoring.

**Methods:**

In this preliminary study, we propose an approach for OSAS monitoring using BCG signals. Applying fast change-point detection to first isolate apnea-suspected episodes would allow for processing only those suspected episodes for further feature extraction and OSAS severity classification. This can reduce both the data to be stored or transmitted and the computational load. Furthermore, our approach directly extracts features from BCG signals without employing a complex algorithm to derive respiratory and heart rate signals as often done in literature, further simplifying the algorithm pipeline. Apnea-hypopnea index (AHI) is then computed based on the detected apnea events (using a random forest classifier) from the identified apnea-suspected episodes. To deal with the expected underestimated AHI due to missing true apnea events during change-point detection, we apply boundary adjustment on AHI when classifying severity.

**Results:**

Cross-validated on 32 subjects, the proposed approach achieved an accuracy of 71.9% for four-class severity classification and 87.5% for binary classification (AHI less than 15 or not).

**Conclusion:**

These findings highlight the potential of our proposed BCG-based approach as an effective and accessible alternative for continuous OSAS monitoring.

## 1 Introduction

Sleep disordered breathing is a group of pervasive and serious sleep disorders characterized by various forms of breathing abnormalities during sleep, including obstructive sleep apnea syndrome (OSAS), hypoventilation, and associated decreased oxygen saturation. OSAS affects about 9–38% of adults worldwide, and this percentage is higher in the obese population (Senaratna et al., 2017). OSAS not only influences the quality of sleep, but is also associated with a variety of serious health issues such as hypertension, cardiovascular disease, stroke and diabetes (Young et al., 2002).

The full night polysomnography (PSG) recording is commonly regarded as the gold standard for the diagnosis of OSAS, as it can record a wide range of multi-channel physiological parameters in detail, including electroencephalogram (EEG), electrooculogram (EOG), electromyogram (EMG), electrocardiogram (ECG), respiratory flow, respiratory effort, and oxygen saturation, etc. (Berry et al., 2012). These parameters enable a comprehensive assessment of a patient’s sleep status and the level of respiratory disturbance. However, PSG recording needs to be performed in a specialized sleep laboratory in a hospital using specialized clinical equipment that is complex and costly to set up by experts, and also requires patients to be monitored overnight in the hospital. This unnatural sleep environment may lead to patient discomfort, which may affect the test results (Bruyneel and Ninane, 2014).

To overcome the limitations of PSG, portable and non-obtrusive sleep monitoring technologies based on cardio-respiratory signals, for example, wrist-worn photoplethysmography and bed-based ballistocardiography (BCG), are gradually attracting more interest (Radha et al., 2021; Haghi et al., 2024; Yoon and Choi, 2023). Portable devices are able to be used in the home setting, which allows patients to be monitored in a more familiar sleeping environment. These home-based devices are usually easier to operate and less expensive than clinical devices. Although these portable, non-obtrusive devices usually record, compared with PSG, fewer physiological parameters, they have been shown able to detect OSAS (Mendonça et al., 2018). In recent years, research on OSAS has made significant progress, especially in terms of monitoring and automated assessment of OSAS using a non-invasive device assisted with machine learning algorithms (Bazoukis et al., 2023; Retamales et al., 2024). For example, the use of a bed-based sensor to measure BCG allows for detection of small vibrations caused by a patient’s cardiac and respiratory activities, and these activities have been demonstrated to associate with sleep apnea, making them possible to be used for OSAS monitoring (Kholghi et al., 2022). BCG is a non-invasive technique measuring the (changes in) mechanical contraction forces generated mainly by the beating heart due to the blood ejection at each cardiac cycle from heart to arteries. In addition, forces generated by gross body movements as well as thoracic and abdominal movements when breathing are also captured by the BCG signal. Different types of sensors can be used to measure BCG such as pressure sensors, optical sensors, accelerometers, and piezo-electric sensors (Joshi et al., 2018). This technique, compared with wearables, is completely non-obtrusive and does not require the patient to wear a sensor, greatly enhancing patient comfort and compliance during nighttime sleep. In addition, BCG devices allow for long-term monitoring, providing an opportunity for continuous OSAS assessment.

Over the past few years, multiple studies have applied BCG to the field of non-obtrusive OSAS monitoring and machine learning models have been developed to automatically detect apneic events and/or classify OSAS severity of patients (Wang et al., 2017; Huysmans et al., 2019; Sadek et al., 2020). Promising performance for apnea detection has been achieved in some studies, even better than that obtained using standard respiratory and/or electrocardiography signals (Olsen et al., 2020; Xie et al., 2024), though comparing OSAS classification performance on different study cohorts is always difficult. While cross validation was applied in some of those studies, it is unclear whether that was strictly “subject-independent” – data from same subjects were either in the training or in the test set. Having a model trained on the data from a subject already exposed to test data from the same subject would result in bias, and therefore such model is impractical and its performance is overrated. In addition, many of those studies require precise identification of heartbeats from BCG signals to characterize heart rate variability using algorithms that are relatively computationally intensive such as template matching (Shi et al., 2008), wavelet transform (Sadek and Abdulrazak, 2021), and even machine learning (Bruser et al., 2011) or the combination of them (Mou et al., 2023). Some other studies employed advanced deep neural network models operated directly on BCG signals (Cimr and Studnička, 2020). However, high computational cost would practically limit the deployment or implementation of those algorithms on a device, particularly for the sake of cost-effectiveness from a commercial perspective.

Based on non-obtrusive BCG signals, the aim of this study was to detect apneic events using a “cost-effective” approach and thereafter classify OSAS severity (normal, mild, moderate, and severe) from the estimated apnea-hypopnea index (AHI). The cost-effective approach was heuristically explored, focusing on using algorithms that require lower computational complexity and reduced intermediate data storage, making the approach more adaptable for implementation on devices with often limited data processing and storage capabilities. To achieve this, we first identified apnea-suspected episodes using a simple change-point detection algorithm instead of applying feature extraction and machine learning apnea detection to the entire recording for apnea detection. Afterwards, we extracted features only from the BCG signal without identifying heartbeats from BCG that would likely require a computationally intensive algorithm. A fast and explainable tree-based machine learning model was used to detect apneic events from all apnea-suspected episodes. Note that the term “apneic events” presented in this paper is also sometimes called “sleep-disordered breathing events” covering obstructive, central, and mixed apnea as well as hypopnea, but excluding other sleep-related breathing disorders such as hypoventilation and hypoxemia.

## 2 Data collection and subjects

**Figure 1:**
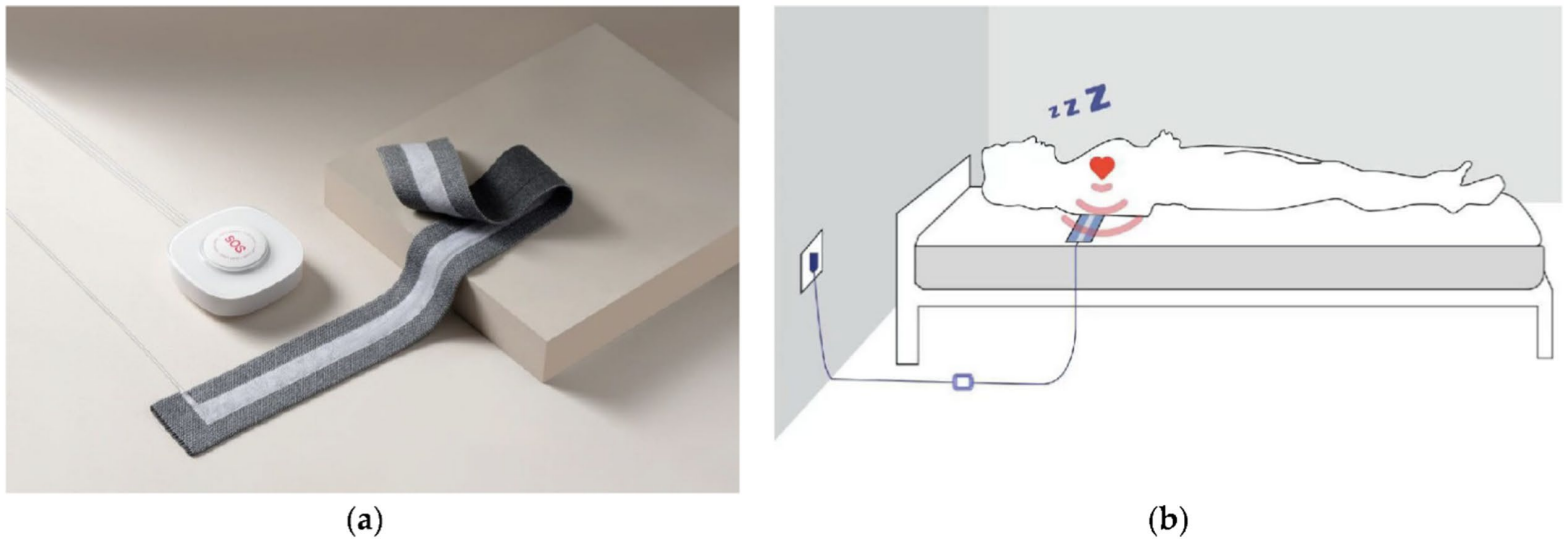
The BCG system **(a)** where a piezoelectric sensor stripe connected to a unit including a microcontroller and a signal conditioning circuit, and the schematic graph **(b)** illustrating the sensor stripe placed under a subject measuring BCG data from the subject during sleep (Su et al., 2022).

As shown in Figure 1a, the non-obtrusive BCG system (Slaap Lekker Monitor, Bobo Technology Ltd., Jiaxing, China) used for data collection is the same as that used in previous studies (Su et al., 2022; Su et al., 2024). The system consists of primarily three modules: a microcontroller, a signal conditioning circuit including amplifier, filter and analog-to-digital converter, and a flexible bed-based piezoelectric sensor stripe (width: 7 cm, length: 72 cm). A digital automatic gain control circuit is designed in the system aiming to adapt the signal amplification to avoid, for example, insufficient signal magnitude or excessive signal magnitude leading to signal clipping (outside the dynamic range). This allows the signal acquisition to fit various conditions that can depend on, for example, the mattress upon which the sensor stripe is placed, the weight of the subject, and changes of sleeping posture. When recording, the sensor stripe was placed above the mattress and underneath the bedsheet as illustrated in Figure 1b.

In this study, BCG data were analyzed from 32 subjects, who were monitored at the Sir Run Run Shaw Hospital (SRRSH), affiliated with the Zhejiang University School of Medicine, Hangzhou, China. The collected BCG signals had a sampling rate of 125 Hz. On average, 8.5 h of BCG signals were recorded per subject during overnight sleep. During BCG monitoring, the subjects underwent PSG monitoring simultaneously, and from the recorded PSG, apneic events (including obstructive apnea, central apnea, mixed apnea, and hypopnea) and sleep–wake states were manually scored by three trained sleep experts with majority vote in case of disagreement. Apnea-hypopnea index (AHI, the number of apneic events per hour during sleep, excluding wake state) was calculated for each patient. The cohort for this study included 8 patients with severe (AHI ≥ 30 per hour), 9 with moderate (15 ≤ AHI < 30 per hour), and 7 with mild (5 ≤ AHI < 15 per hour) OSAS. The remaining 8 subjects had no OSAS diagnosed (AHI < 5 per hour). The included patients had no (prior) OSAS-related therapy (e.g., medication or continuous positive airway pressure) nor other diagnosed severe comorbidities. Among the patients, one had atrial fibrillation and five had hypertension where four were taking antihypertensive drugs. All subjects provided an informed consent form, and the study was approved by the local ethics committee of the SRRSH. On average, the subjects included in this study were dominated with obstructive (57.4%) and hypopnea (32.9%) events. Details of subjects and their OSAS characteristics are provided in Table 1.

**Table 1:** Subject and OSAS characteristics (*N* = 32).

Characteristics	Mean (SD) over subjects or count
Gender	27 males, 5 females
Age	49.5 (14.3) years
Body mass index	25.5 (4.6) kg/m^2^
Normal (AHI < 5)	8
Mild (5 ≤ AHI < 15)	7
Moderate (15 ≤ AHI < 30)	9
Severe (AHI > 30)	8
Percentage of obstructive events	57.4% (22.9%)
Percentage of central events	1.8% (6.2%)
Percentage of mixed events	4.8% (7.3%)
Percentage of hypopnea events	32.9% (23.1%)

The estimated prevalence of OSAS significantly varied across studies, age groups and countries. It was estimated to be 12.6% in middle-aged adults (Wei et al., 2022) which are considered the population of interest for home-based OSAS screening since many of them are likely underdiagnosed. Due to the proof-of-concept scope of this study, we accepted a lower confidence level at 90% and a higher margin of error at 10% (Fabozzi et al., 2023) for sample size calculation, leading to a minimum sample size of 30. We therefore considered the chosen sample size of 32 acceptable in power (albeit not strong) in this preliminary study.

## 3 Methods

**Figure 2:**
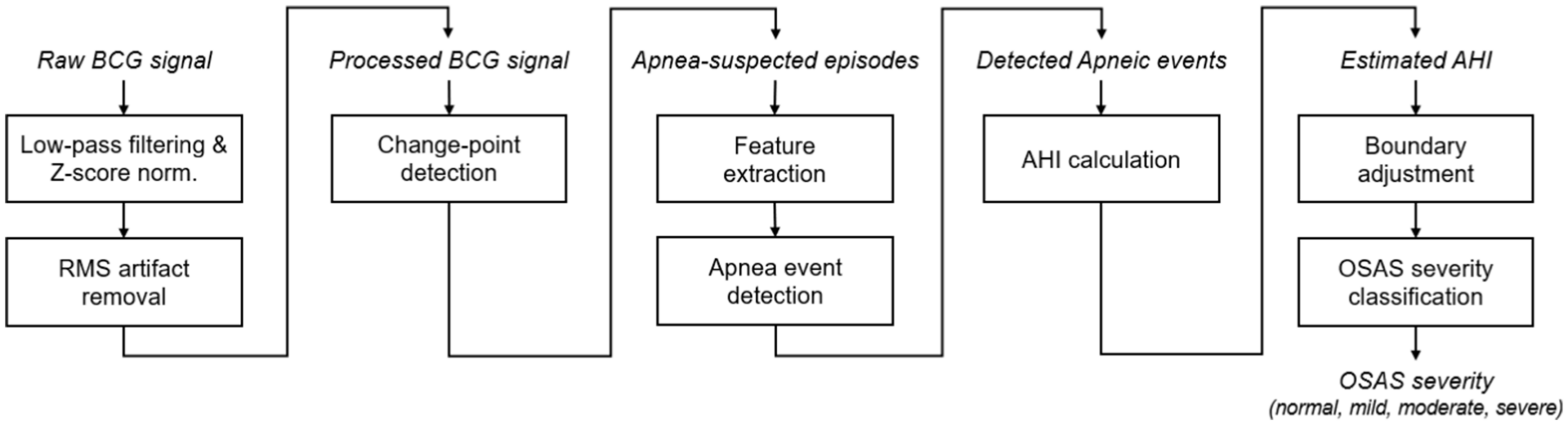
Flowchart of the proposed approach for OSAS monitoring, including, from left to right, BCG signal processing, change-point detection, pre-identification of apnea-suspected episodes, AHI estimation, and OSAS severity classification. RMS, root mean squired filtering.

The flowchart of the proposed approach for OSAS monitoring and assessment in this preliminary study is shown in Figure 2, where the key steps will be explained in detail in the following subsections.

### 3.1 Signal processing and labeling

The acquired BCG signals were firstly processed using a 7th-order band-pass Butterworth filter (0.05–3 Hz) to suppress baseline wander and motion artifacts while preserving cardio-respiratory information. The processed signal was then z-score normalized and further processed with a root mean square (RMS) filter to further remove motion artifacts. Specifically, the sliding window of the RMS filter was empirically set as 30 s and the signal segments where the RMS amplitude exceeded one SD from the mean were identified as motion artifacts and therefore discarded, under the assumption that no apneic events occurred during these movement periods.

**Figure 3:**
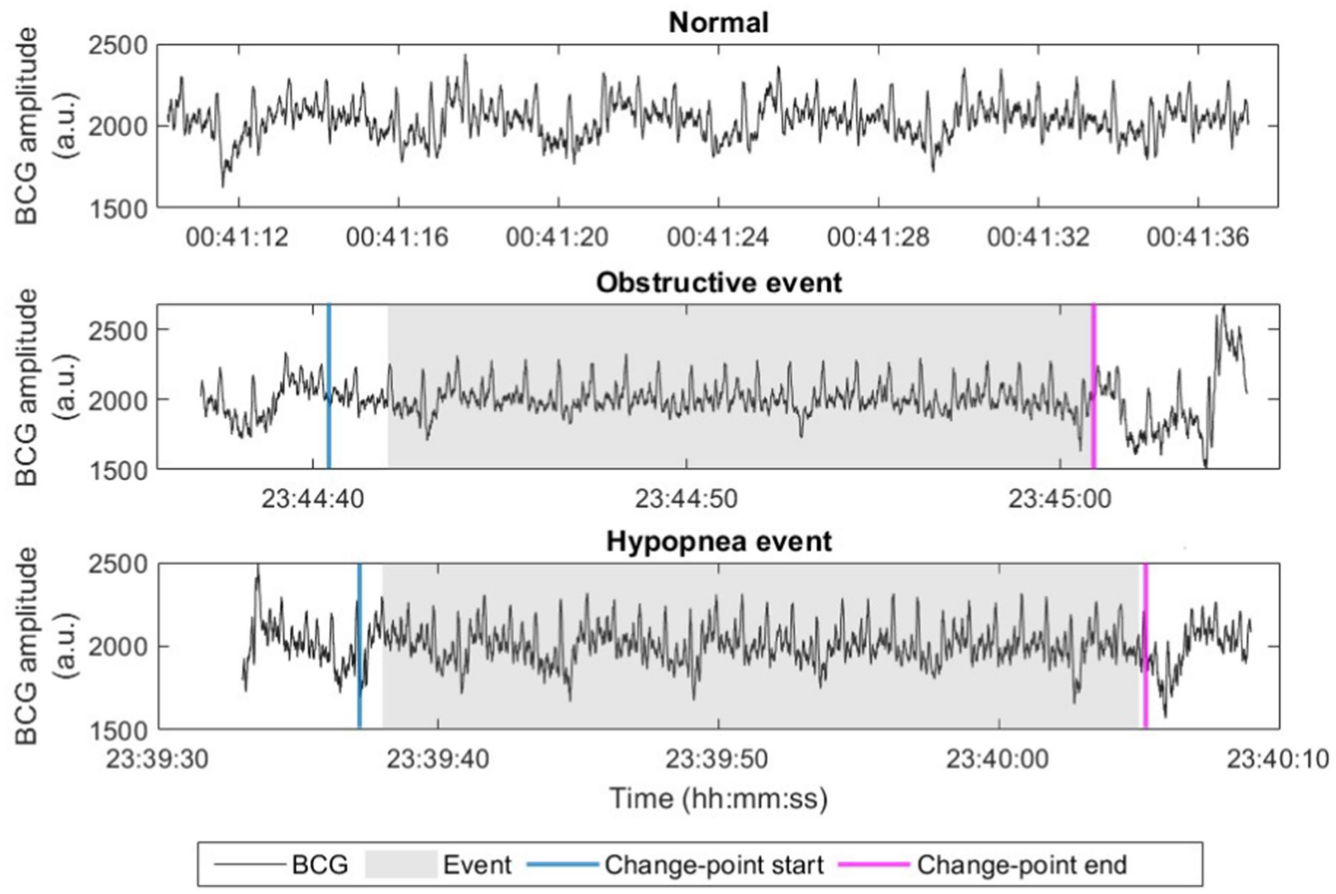
Examples of raw BCG signals (in arbitrary unit, a.u.) with normal breathing, obstructive event, and hypopnea event. Start and end points of the apnea-suspected episode after change-point detection (change-point start and change-point end) are marked.

To efficiently identify potential apneic events without applying machine learning to the full night recording, we applied a fast change-point detection algorithm using the MATLAB function *findchangepts* (Lavielle, 2005; Killick et al., 2012), which provided a coarse identification of apnea-suspected episodes. Standard deviation (SD) of the signal was empirically chosen to measure the changes and the threshold was set as 50 to identify these suspected episodes. The episodes identified less than 10 s too short to be apneas or hypopneas were excluded for further detection. The remaining episodes were then classified using a machine learning model as either apneic or non-apneic events for final AHI estimation. For labeling purpose, the start and end timestamps of each true apneic event were first annotated based on the synchronously collected PSG signals by sleep experts. The apnea-suspected episodes were labeled as positive (1) if they overlaps with an annotated true apneic event, and negative (0) otherwise. Figure 3 compares a normal BCG signal (without apnea), a signal including an obstructive event, and a signal including a hypopnea event, where breathing-related (low frequency) fluctuation modulated in signal (peak) envelope can be observed in the normal BCG signal while this is less visible with slightly reduced envelope fluctuation in the hypopnea event and is almost invisible in the obstructive event. Besides, the start and end of the detected change points are indicated in the figure, overlapping with both true events.

### 3.2 Feature extraction

We generated in total 38 features from the processed BCG signals for each apnea-suspected episodes after change-point detection. Since the suspected episodes can vary in length, features should be insensitive to signal length. Table 2 summarizes all the features extracted in the time and frequency domains.

**Table 2:** Description of BCG features used for OSAS monitoring.

Features	Domain	Description
Pow, Sd, Zcr	Time	Signal power, SD, and zero-crossing rate
Kurt, Skew	Time	Signal kurtosis and skewness
TEmean, TEsd	Time	Mean and SD of signal Teager energies
SampEn	Time	Sample entropy of signals
Int, Slope, Res	Time	Intercept, slope, and residual of linear fitting on signal
AR1, AR2, …, AR15	Time	Coefficients of autoregressive modeling on signal
DF1, DF2	Frequency	Dominant frequency between 0 and 0.5 Hz (1) and 0.8–1.8 Hz (2)
SP1, SP2	Frequency	Spectral powers of dominant frequency DF1 and DF2
SPLmean, SPLsd	Frequency	Mean and SD of spectral powers between 0 and 3 Hz
SPHmean, SPHsd	Frequency	Mean and SD of spectral powers between 3 and 10 Hz
VLF	Frequency	Spectral power in very low frequency band (0–0.05 Hz)
LF	Frequency	Spectral power in low frequency band (0.05–0.15 Hz)
HF	Frequency	Spectral power in high frequency band (0.15–0.5 Hz)
LF/HF	Frequency	Ratio between LF and HF

For time-domain BCG features, the signal power and SD were computed and these features might be associated with breathing amplitude and motion artifacts. Zero-crossing rate indicates the dominant frequency of a signal (Sadek et al., 2019). More assertive distribution-related metrics such as kurtosis and skewness of the signal were extracted (Huysmans et al., 2019). Teager energy operator collectively quantifies high-resolution time and frequency energy (Joshi et al., 2018), and the mean and SD of Teager energy analyzed its average and changes over time. Sample entropy to assess signal complexity or regularity has been applied for BCG-based OSAS identification in a previous study (Zhao et al., 2015). Intuitively, the presence of an apneic event may correlate a decreasing trend in signal amplitude due to decreased or cessation of breathing, we therefore tried to characterize such trend using a simple linear fitting model with parameters including the model’s intercept, slope, and residual. To further characterize the signal waveform structure, a 15th-order autoregressive (AR) model was applied (Alivar et al., 2019), resulting in 15 AR coefficients used as features.

As mentioned before, when a subject is sleeping, the BCG sensor measures the vertical force generated by the subject’s heartbeat and breathing. Hence the dominant frequency (the peak frequency in the Fourier-transformed power spectrum) between 0.8 and 1.8 Hz and between 0 and 0.5 Hz and its spectral power were obtained, where presumably these two dominant frequencies corresponded to average heart rate and respiratory rate of the signal, respectively. Mean and SD of the normalized spectral power between 0 and 3 Hz and between 3 and 10 Hz were used to approximate characteristics corresponding to major cardio-respiratory activity and noise such as motion artifact and “I-J-K” complex harmonics (Wiard et al., 2010; Inan et al., 2009), respectively. Inspired by the analyses of breathing rate and heart rate variability (Wang et al., 2017; Huysmans et al., 2019; Fonseca et al., 2015), we computed the normalized spectral power in the very low frequency band (VLF, 0–0.05 Hz), low frequency band (LF, 0.05–0.15 Hz), and high frequency band (HF, 0.15–0.5 Hz), and also the ratio between LF and HF powers.

### 3.3 Modeling and evaluation

Random forest (RF) is an efficient, explainable machine learning algorithm with inherent capability of selecting features or ranking feature importance when making classification decision (Breiman, 2001), and it has proven successful in automatically assessing OSAS (Wang et al., 2017; Balci et al., 2022). We hence employed an RF classifier with features extracted from solely BCG signals to detect true apneic events from all pre-identified apnea-suspected episodes, i.e., to classify all suspected episodes as apneic and non-apneic events. For comparison, we also applied other classifiers with different mechanisms including support vector machines (SVM) and logistic regression (LR).

Model performance in detecting apneic events and estimating AHI was evaluated using leave-one-out cross-validation. Specifically, the data was partitioned into 32 folds, each corresponding to a subject. In each iteration, features from one subject served as the test set, while the model was trained on features from the remaining 31 folds which formed the training set. Within each training set of 31 subjects during each cross-validation iteration, the data were further partitioned into training and validation sets where the validation set was used for parameter optimization. For validation, four subjects – one from each OSAS severity category – were randomly selected in each iteration of cross-validation. Key model parameters, including the number of trees, minimum leaf size, and prior probability, were optimized using Bayesian optimization based on validation performance, and then the trained model with optimized parameters was applied to the test data from the left-out test subject. The detected apneic events for each test subject by the model were then used to estimate the AHI for that subject. Model performance was assessed by comparing detected apneic events with PSG-based (true) apneic events annotated by sleep experts, and we calculated aggregate or overall sensitivity, precision, and F1 score pooling the detection results from all subjects. Scatter and Bland–Altman plots were used to visualize AHI estimation and the Spearman’s correlation was examined between the (BCG-based) estimated AHI and the corresponding (PSG-based) actual AHI values.

For comparison purposes, we also extracted features from respiratory and heart rate variability (i.e., heartbeat interval) signals derived from BCG signals, similar to previous studies (Long et al., 2014; Wang et al., 2017; Zhao et al., 2015). The detection performance achieved using solely the BCG features proposed in this work (i.e., features extracted directly from BCG signals) was compared with that using the combination of BCG and BCG-derived respiratory (BDR) features, and that of BCG, BDR, and BCG-derived heart rate variability (BDH) features. Detailed descriptions of BDR and BDH features are provided in the Supplementary Tables S1, S2. These features were extracted aiming to quantify cardio-respiratory dynamics and morphological properties that can be informative to further capture OSA-related characteristics when apneic events are present.

For classification of OSAS severity, an underestimated AHI would be expected primarily likely because of two possible reasons. First, some apneic events might be missed after change-point detection. The missed apneic events might be more associated with hypopneas since hypopneas were mainly defined as slowed, shallow, and restricted breathing, whose change points were, intuitively, more difficult to be accurately detected, rather than apneas with complete cessation of breathing. Second, the estimated AHI was computed based on total recording time that was actually respiratory event index (REI) while the actual PSG-based AHI was computed based on total sleep time. Therefore we decided to adjust the potential AHI underestimation by changing the three thresholds or boundaries (normal-mild: 5, mild–moderate: 15, and moderate–severe: 30) when classifying OSAS severity. The boundaries were optimized on the training data using grid search aiming at maximizing OSAS severity classification performance, resulting in different boundary values during different iterations or folds of cross-validation. A unique set of boundaries was assembled by, for each boundary, taking the median of the optimized or adjusted boundary values over cross-validation folds. This new set of boundaries were then used to determine OSAS severity for all subjects.

Confusion matrix, overall accuracy, and Cohen’s kappa coefficient were obtained to assess the model’s performance in classifying OSAS severity. Results with and without boundary adjustment were compared. Results in distinguishing between patients with normal to mild (AHI < 15) and moderate to severe (AHI ≥ 15) were also reported. We also investigated the correlation between underestimated AHI and hypopnea index (number of hypopnea events per hour, calculated as the total number of hypopnea events divided by total recording time in hours per night per subject).

## 4 Results

A total of 6,320 apnea-suspected episodes were pre-identified after change-point detection, where 3,032 episodes were associated with true apneic events (out of the total number of 5,057 true apneic events) when compared with PSG-based annotation. All the 6,320 episodes were then included for further feature extraction and classification between apneic and non-apneic events using leave-one-out cross-validation and the RF classification algorithm. The overall apneic event detection results on the entire recordings are shown in Table 3. When using only the BCG features, an overall or aggregate sensitivity of 0.33, a precision of 0.57, and an F1 score of 0.42 were reached. It can be seen that in total around 67% true apneic events were missed, where 59% of them (accounting for 40% of total apneic events) were already missed by change-point detection and the remaining ones (accounting for 27% of total events) were missed by the subsequent machine learning model. Notably, these results were only slightly lower than those when combining BCG features with cardio-respiratory features extracted from BCG-derived respiratory and heart rate variability signals. In general, the apneic event detection results achieved using RF were better than those using SVM and LR as presented in Supplementary Tables S3, S4, respectively, in particular when using the BCG features solely.

**Table 3:** Performance of apneic event detection.

	BCG features	BCG, BDR features	BCG, BDR, BDH features
Sensitivity	0.33	0.35	0.34
Precision	0.57	0.56	0.63
F1 score	0.42	0.43	0.44

**Figure 4:**
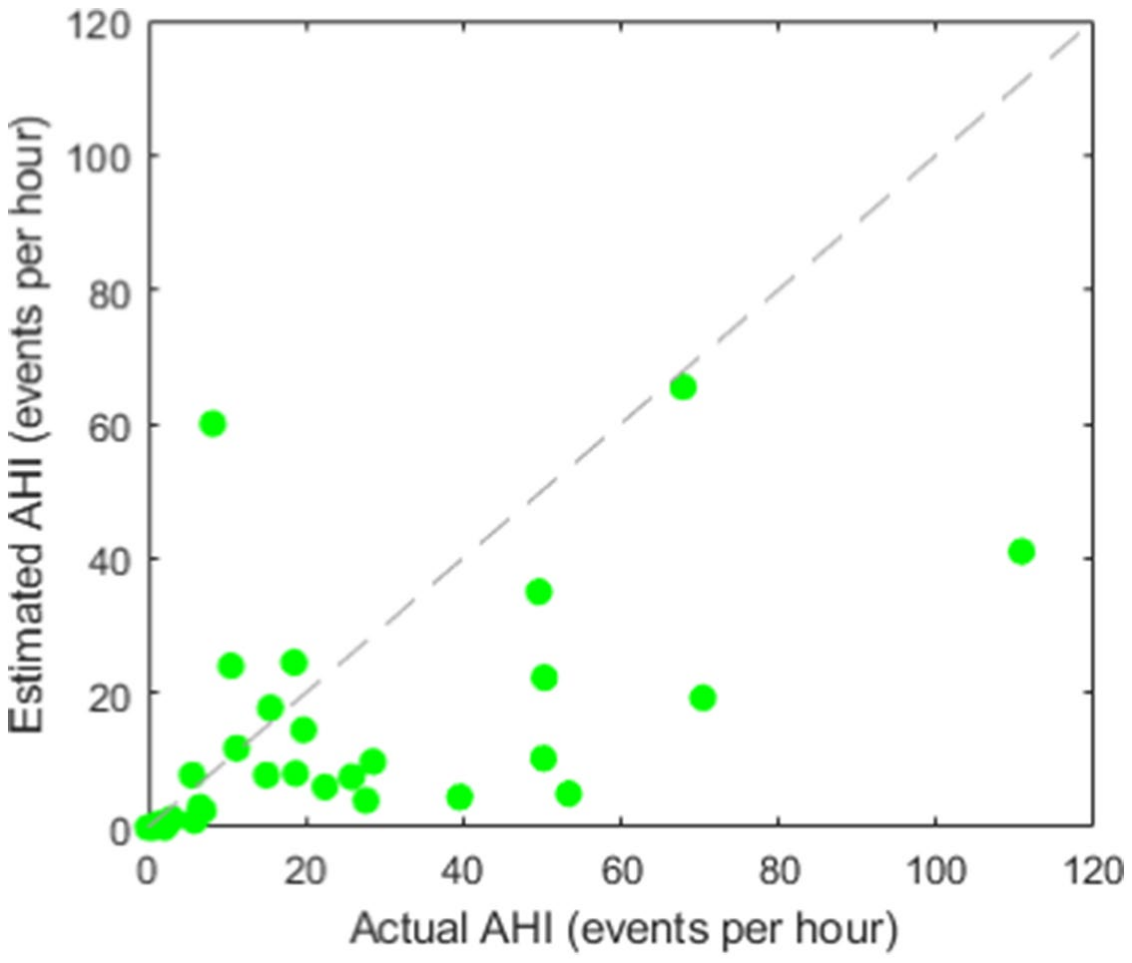
Scatter plot for BCG-based AHI estimation (correlation coefficient: *r* = 0.73).

**Figure 5:**
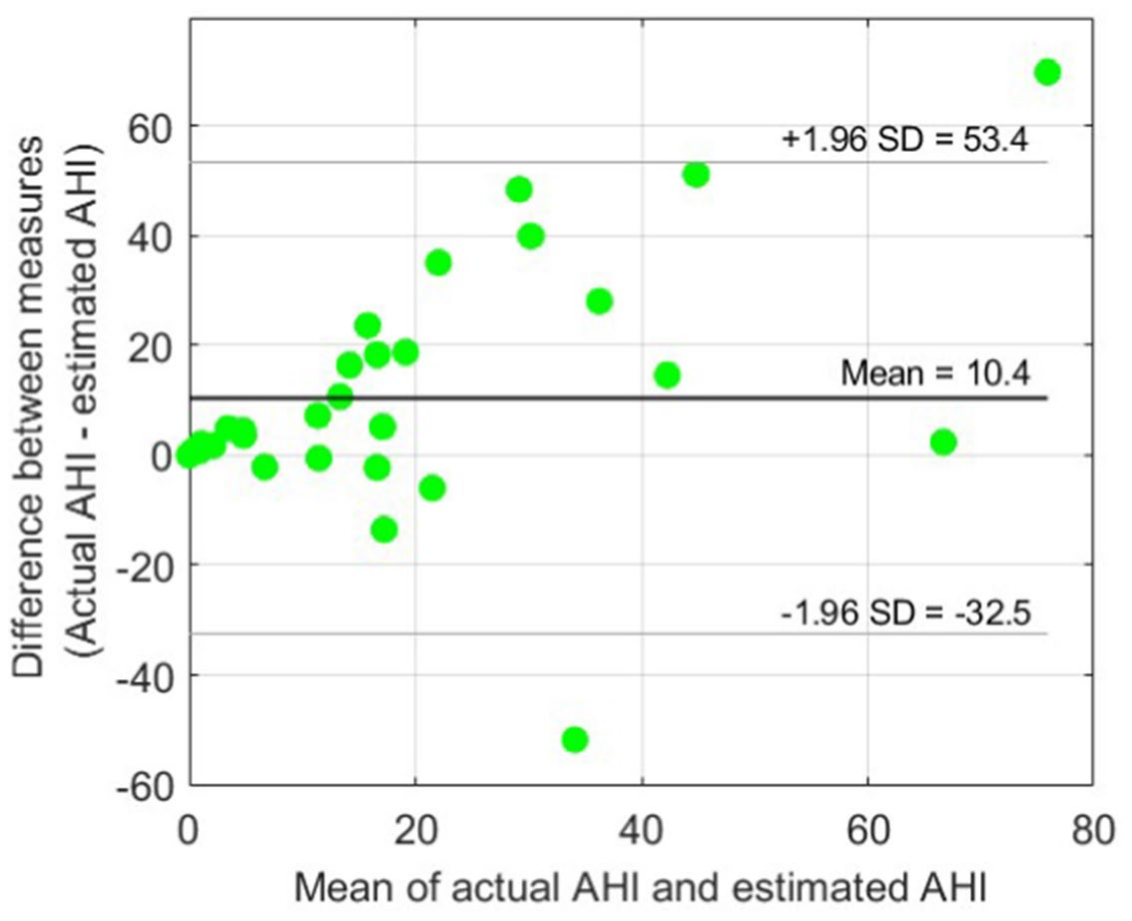
Bland–Altman plot for BCG-based AHI estimation. An average underestimation of AHI = 10.4 events per hour can be observed compared with the reference actual AHI. The 95% limits of agreement (± 1.96 SD) are also indicated.

Figure 4 shows the scatter plot for AHI estimation using only BCG features. A relatively high Spearman’s correlation coefficient *r* of 0.73 was achieved. However, as we expected, the BCG-based AHI values obtained using our model were strongly underestimated compared with the actual PSG-based AHI values. This can also be seen in the Bland–Altman plot in Figure 5, showing an average underestimation of 10.4 events per hour in AHI.

**Figure 6:**
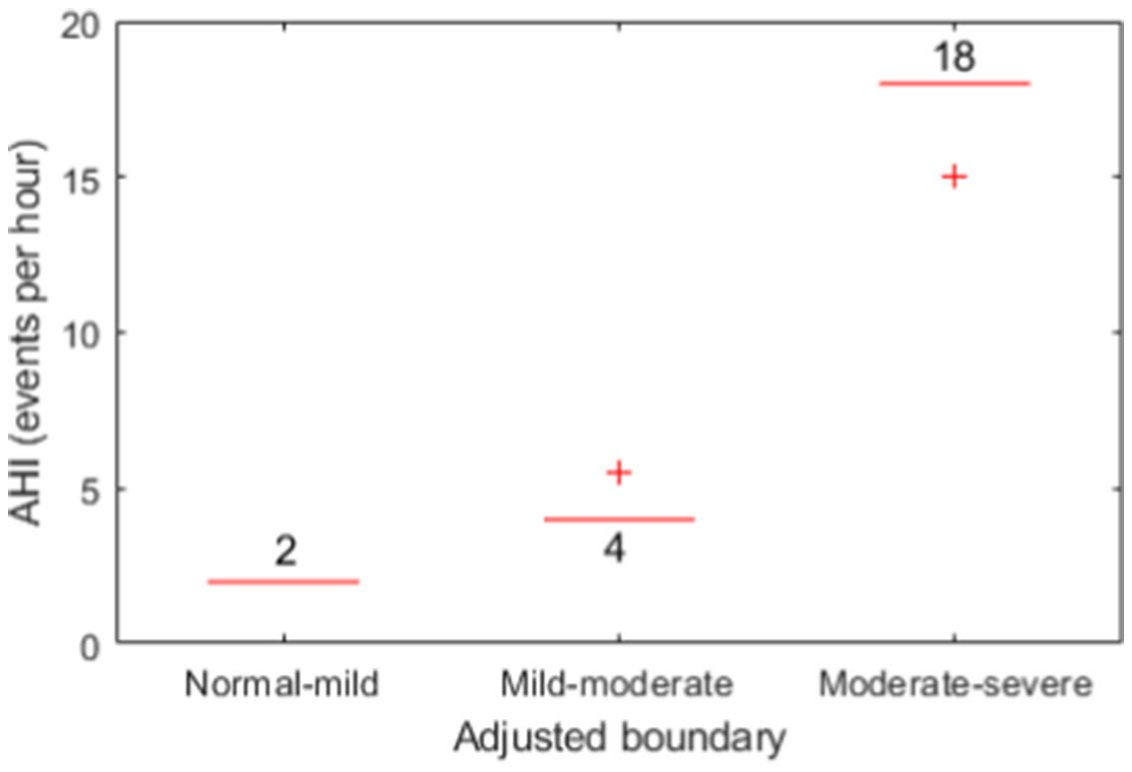
Boxplots of the three adjusted boundaries used for OSAS severity classification for different subjects.

The confusion matrices for OSA severity classification before and after adjustment of boundaries are presented in Tables 4, 5, respectively. The adjusted boundaries were 2 for normal-mild, 4 for mild–moderate, and 18 for moderate–severe, which means that a subject was classified without OSAS when AHI < 2, with mild OSAS when 2 ≤ AHI < 4, moderate OSAS when 4 ≤ AHI < 18, and with severe OSAS when AHI > 18. These boundaries were lower than the original ones (5, 15, and 30), demonstrating the presence of underestimation of AHI using the machine learning model. Figure 6 shows the boxplots of adjusted boundaries across cross-validation folds for different subjects. This figure indicates that the adjusted boundaries were mostly consistent for different folds, except for a couple of “outliers.”

**Table 4:** Confusion matrix for BCG-based OSAS severity classification before boundary adjustment.

		Predicted class*			
		Normal	Mild	Moderate	Severe
True class	Normal	8	0	0	0
	Mild	3	2	1	1
	Moderate	1	6	2	0
	Severe	1	2	2	3

^*^OSAS severity classification criteria with original boundaries: AHI < 5 (normal), 5 ≤ AHI < 15 (mild), 15 ≤ AHI < 30 (moderate), AHI ≥ 30 (severe). Correct classifications are indicated with green shading.

**Table 5:** Confusion matrix for BCG-based OSAS severity classification after boundary adjustment.

		Predicted class*			
		Normal	Mild	Moderate	Severe
True class	Normal	8	0	0	0
	Mild	1	2	2	2
	Moderate	0	0	8	1
	Severe	0	0	3	5

^*^OSAS severity classification criteria with adjusted boundaries: AHI < 2 (normal), 2 ≤ AHI < 4 (mild), 4 ≤ AHI < 18 (moderate), AHI ≥ 18 (severe). Correct classifications are indicated with green shading.

**Figure 7:**
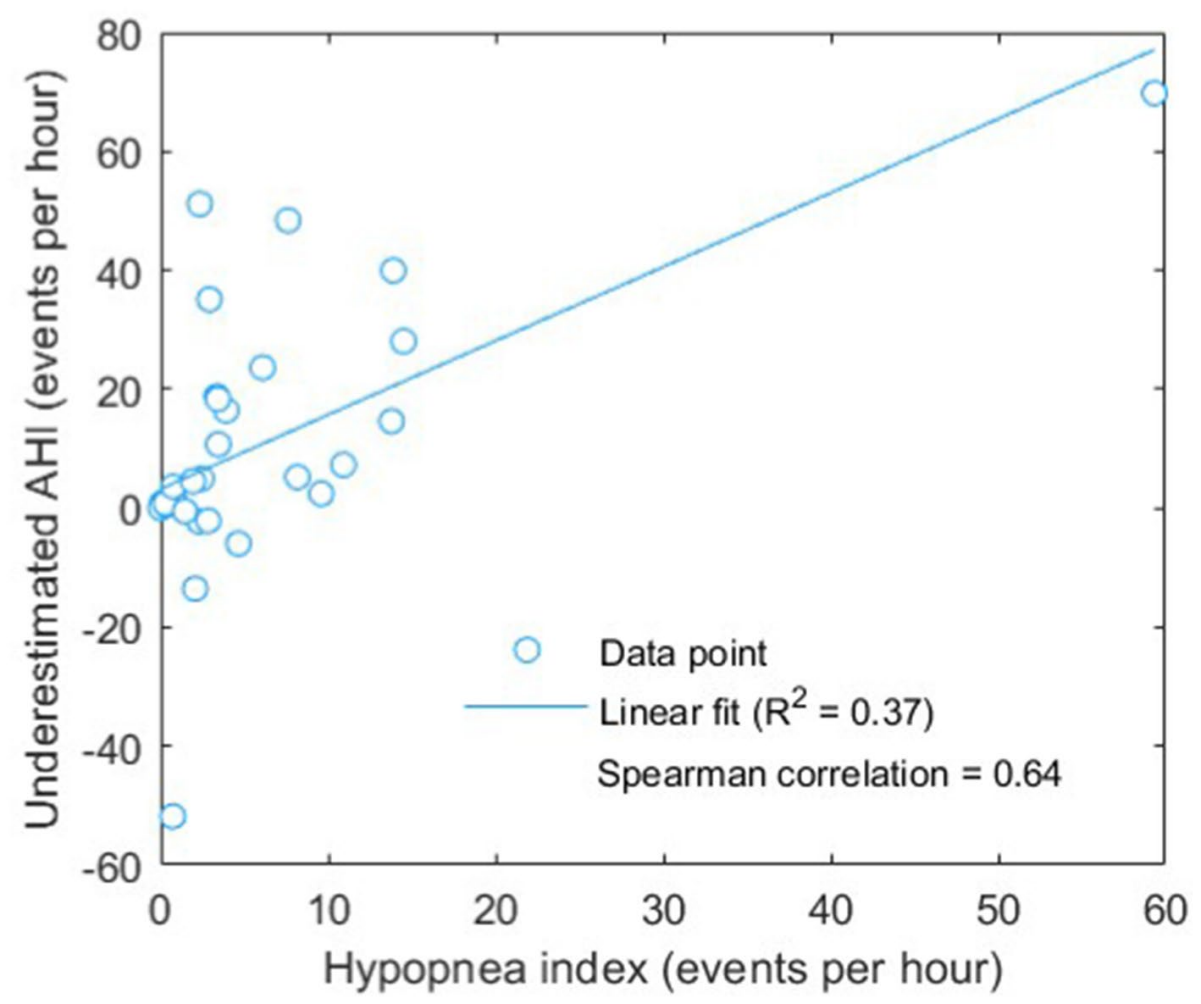
Scatter plot of underestimated AHI (actual AHI – estimated AHI) versus hypopnea index. Linear fitting was done showing a positive correlation, with a (Pearson) *R*^2^ value of 0.37 and a significant (Spearman) correlation coefficient of 0.64 (*p* < 0.001).

As presented in Table 6, the severity underestimated before the adjustment of boundaries showed a poor accuracy and kappa value of 46.9% and 0.30, respectively. After adjustment of the boundaries for severity classification, the performance was largely improved to an accuracy of 71.9% and a kappa of 0.62 indicating a substantial agreement between the actual AHI based on PSG-based annotation and the estimated AHI based on our proposed BCG-based automatic approach. Besides, combining both BCG and BDR and/or BDH features did not yield improvement in classification performance compared with the use of solely BCG features. Considering distinguishing between normal-to-mild and moderate-to-severe OSAS, promising performance has been achieved: accuracy = 87.5%, kappa = 0.75. The scatter plot (Figure 7) shows the relationship between the underestimated AHI (= actual AHI – estimated AHI) and the hypopnea index. A significant correlation was found, with a Spearman’s correlation coefficient of 0.64 (*p* < 0.001).

**Table 6:** Summary of OSAS severity classification results.

	Performance measure	Four class*	Two class**
Before boundary adjustment	Accuracy	46.9%	62.5%
	kappa	0.30	0.27
After boundary adjustment	Accuracy	71.9%	87.5%
	kappa	0.62	0.75

*Four class OSAS severity classification: normal, mild, moderate, and severe. **Two class OSAS severity classification: normal-to-mild and moderate-to-severe.

## 5 Discussion

As presented before, the change-point detection led to a high miss rate of apneic events and also many false positives, though machine learning was expected to correct some of the false detections. Actually the SD threshold used in change-point detection may be overly sensitive to the tradeoff between miss rate and false detections. Further optimization on a large dataset with more patients may be required to verify the effect of this threshold on final OSAS monitoring and assessment. The overall performance in apneic event detection using the RF classifier is similar to that reported in a prior study using a non-unobtrusive BCG-based bed sensor (Sadek et al., 2020) but they performed machine learning-based event detection on a minute-by-minute basis. When comparing different classification algorithms (classifiers) in apneic event detection, RF in general outperformed SVM and LR (Supplementary Tables S1, S2). In addition to the conventional machine learning algorithms, employing a more advanced classification algorithm such as deep neural networks would has the potential to further improve the detection performance. Yet we decided not to investigate this since deep learning usually requires a large, diverse dataset to train a reliable model while in this study only 32 patients’ data were included. Although the computational complexity of training a deep learning model is high, deploying a trained model usually needs much less computational load. Thus it merits further investigation on using advanced deep neural networks for BCG-based OSAS assessment when a much larger dataset is available.

In general, we achieved a good performance in assessing OSAS (i.e., classifying OSAS severity) in this study. Unlike many previous studies requiring complex algorithms to derive respiratory and heart-rate signals from BCG for feature extraction as stated in Section 1, the proposed approach only used features directly extracted from BCG signals showing comparable results in both apneic event detection and OSAS severity classification (Huysmans et al., 2019; Hayano et al., 2024). The BCG-based approach would be potentially suitable for continuous monitoring or screening of OSAS at home, in particular because of its high sensitivity at a relative low expense of specificity (false positives of 4 out of 15) in detecting moderate and severe cases from normal and mild cases as shown in Table 5. Beyond this preliminary study with a small sample size, in the future including more diverse samples (patients) in terms of age, sex, BMI, and OSA severity would help further verify the performance of our proposed approach in OSAS assessment and its generalizability to different patient subgroups with respect to demographics and clinical conditions.

There was a marked number of apneic events missed after the change-point detection before applying machine learning, which would likely be the cause leading to the clear underestimation of AHI values. To compensate for that, adjusting the boundaries for decision-making of OSAS severity has largely improved the severity classification performance, with an accuracy from 46.9 to 71.9% for four classes (normal, mild, moderate, and severe) and from 62.5 to 87.5% for two classes (normal-to-mild and moderate-to-severe). Notably, the adjusted boundaries for each cross validation fold were mostly the same (see Figure 6), demonstrating their consistency over subjects on our dataset, yet this requires to be further verified on an external, larger dataset.

The reduced boundary values (AHI of 2, 4, and 18) also indicate the underestimation of AHI and the improved performance demonstrates the effectiveness of boundary adjustment. Interestingly, the level of the underestimation was found to be significantly correlated to the hypopnea index, implying that hypopnea events were more difficult to be “preserved” or identified by the change-point detection algorithm used in the present study. Actually, this was expected because the changes in BCG signal pattern during hypopnea would be less profound compared with that during other subtypes of apneic events with complete cessation of breathing (see Figure 3 as an example). Such underestimation would lead to the dependency of optimal boundaries for OSAS severity classification on the characteristics of patients such as hypopnea index for example. In this regard, future study should either improve the change-point detection to be robust or adaptive to different subtypes of apneic events or explore adaptive boundaries that are sensitive to hypopnea index. In addition to hypopnea index, other confounding factors or characteristics that might affect the boundaries are worth further analysis. For example, because the estimated AHI was computed based on total recording time instead of total sleep time (which is actually REI as mentioned before), a lower sleep efficiency should correspond to a stronger underestimation of AHI. Due to the unavailability of sleep efficiency in our dataset, improving boundary adjustment by incorporating sleep efficiency should be studied in the future.

Comparing model performance with previous works in literature is often hard, mainly due to differences in, for example, sample size, patient and OSAS characteristics included, and training/testing procedure. Nevertheless, the results obtained in our study are found to be comparable to some recent studies using traditional ECG and respiratory effort or using other obtrusive sensors such as abdominal/thoracic belt and pulse oximetry for OSAS severity classification (accuracy of ~70%) (Retamales et al., 2024; Xie et al., 2024). Though our performance was lower than a previous BCG-based model for classifying OSAS severity (Wang et al., 2017), the composition of apnea subtypes (primarily apnea and hypopnea) in the dataset used in that study is unclear. Model performance in OSAS assessment has been shown to be dependent of the subtypes (Balci et al., 2022). A reported accuracy of >94% in that study might be considered over-optimistic which is already higher than the result using pulse oximetry, thoracic and abdominal movement from PSG (Retamales et al., 2024).

Regarding the implementation considerations of the proposed approach, it is important to note that, for home monitoring health service providers (home-based OSAS monitoring in this case), implementing algorithms or models in the device is often considered advantageous in practice. This can avoid subscribing expensive cloud computing services for streaming and processing data from an increasingly large number of end users, while integrating an advanced high-performance processing unit in the device to run complex algorithms would drastically increase the cost. Hence, developing simpler algorithms requiring less floating-point operations and intermediate data storage that can be implemented in a lower-cost processing unit is crucial. Nevertheless, in the future the performance and usability of the proposed BCG-based OSAS assessment approach should be further validated outside of a hospital setting, in a home study.

A major limitation of our study was the use of a small dataset, although it is a preliminary, proof-of-concept study aimed at demonstrating the feasibility of the proposed approach for BCG-based OSAS monitoring. Leave-one-out subject-independent cross validation was employed to avoid bias and/or overfitting when training and parameter optimization. However, the model and the parameters might still be overfitted to this small dataset, mainly in terms of sample size (i.e., number of patients). It is therefore unclear how the model would generalize to other patients, promising its further validation on external, larger datasets in future work. In addition, although the boundary adjustment led to improved performance in OSAS severity classification, the adjusted boundaries might also overfit to the patients included in this study and they might not be optimal for new, unseen patients. The factors sensitive to the optimal boundaries are still unclear or undetermined, posing a risk of uncertainty in using those adjusted boundaries reported in this paper. When a larger dataset with more samples (patients) is available in the future, the possible sensitive factors should be investigated and the uncertainty should be analyzed. Another limitation is the lack of algorithm cost analysis of our proposed approach. The focus of our study was on initially investigating the approach’s feasibility hence we took a heuristic method to first evaluate whether our approach would lead to plausible BCG-based OSAS assessment results if we used relatively simple algorithms or we left out the part requiring the use of complex algorithms. Nevertheless, the computational complexity/cost and data storage of the proposed algorithm pipeline as well as the technical specifications of required processing and storage units should be quantitatively analyzed in the future.

## 6 Conclusion

This preliminary study presents a cost-effective approach for OSAS severity classification using BCG signals. By applying change-point detection and direct feature extraction from BCG signals, the method achieved good classification performance comparable to existing studies but with a lower computational complexity. Although several limitation exists, the results demonstrated the feasibility of using our proposed BCG-based approach for effective and accessible OSAS assessment, offering a promising solution for home monitoring of OSAS.

## Data Availability

The datasets presented in this article are not readily available because data cannot be shared publicly due to ethical restrictions. The anonymized summary data may be accessible, but the accessibility depends on whether it poses a risk of patient identification. Requests to access the datasets should be directed to Peilin Lu, zxa2004@zju.edu.cn.
