# Supplementary material for: Non-obtrusive monitoring of obstructive sleep apnea syndrome based on ballistocardiography: a preliminary study
Source: Front Neurosci. 2025 Mar 20;19:1549783. doi: 10.3389/fnins.2025.1549783 (PMC11965354; doi:10.3389/fnins.2025.1549783)
Supplement: Supplementary file 1 [file Table_1.docx]

Supplementary Material

# BDR and BDH features

For apneic event detection, there were 16 features extracted BCG-derived respiratory (BDR) signals and 18 from BCG-derived heart rate variability (BDH) signals for each pre-identified apnea-suspected episode, as described in Supplementary Table S1 and S2, respectively.

**Supplementary Table S1. Description of BDR features.**

| **Feature** | **Description** |
| --- | --- |
| rSd | Standard deviation of the signal |
| rSampEn | Sample entropy of the signal |
| rPnum | Number of peaks of the signal |
| rVnum | Number of valleys of the signal |
| rRateP | Respiratory rate estimated as the number of peaks divided by episode length |
| rRateV | Respiratory rate estimated as the number of valleys divided by episode length |
| rRateSdP | Standard deviation of instantaneous respiratory rate estimated as the inverse of inter-breath intervals between consecutive peaks |
| rRateSdV | Standard deviation of instantaneous respiratory rate estimated as the inverse of inter-breath intervals between consecutive valleys |
| rSdmP | Standardized median (median over inter-quartile range) of respiratory peaks |
| rSdmV | Standardized median (median over inter-quartile range) of respiratory valleys |
| rSampEnP | Sample entropy of the peak sequence |
| rSampEnV | Sample entropy of the valley sequence |
| rBBCPmean | Mean breath-to-breath correlations (breath between consecutive peaks) |
| rBBCPsd | SD of breath-to-breath correlations (breath between consecutive peaks) |
| rBBCVmean | Mean breath-to-breath correlations (breath between consecutive valleys) |
| rBBCVsd | SD of breath-to-breath correlations (breath between consecutive valleys) |

**Supplementary Table S2. Description of BDH features.**

| **Feature** | **Description** |
| --- | --- |
| hHRmean | Mean heart rate |
| hHRmax | Maximum heart rate |
| hHRmin | Minimum heart rate |
| hSDNN | Standard deviation of inter-beat intervals |
| hPNN50 | Percentage of changes in successive inter-beat intervals exceeding 50ms |
| hRMSSD | Root mean square of successive differences between inter-beat intervals |
| hSDSD | Standard deviation of the consecutive beat-to-beat differences |
| hMAD | Mean absolute difference of inter-beat intervals |
| hSampEn | Sample entropy of inter-beat intervals |
| hIBI10 | 10 percentile of inter-beat intervals |
| hIBI25 | 25 percentile of inter-beat intervals |
| hIBI50 | 50 percentile of inter-beat intervals |
| hIBI75 | 75 percentile of inter-beat intervals |
| hIBI90 | 90 percentile of inter-beat intervals |
| hVLF | Normalized spectral power in the very low frequency band (0.0033-0.04 Hz) |
| hLF | Normalized spectral power in the low frequency band (0.04-0.15 Hz) |
| hHF | Normalized spectral power in the high frequency band (0.15-0.4Hz) |
| hLFHF | Ratio between the LF and HF band |

# Apneic event detection

Supplementary Table S3 and S4 present the apneic event detection results using two other classifiers, including support vector machines (SVM) and logistic regression (LR), respectively. Like RF, same leave-one-out cross-validation procedure was applied for SVM and LR. For SVM, Gaussian kernel was used and key parameters such as BoxConstraint were optimized using Bayesian optimization. Similarly, for LR, regularization methods including least absolute shrinkage and selection operator (lasso) and ridge, and the weight of regularization term Lambda were optimized using Bayesian optimization also.

**Supplementary Table S3. Performance of apneic event detection using SVM.**

|  | **BCG features** | **BCG, BDR features** | **BCG, BDR, BDH features** |
| --- | --- | --- | --- |
| **Sensitivity** | 0.34 | 0.34 | 0.34 |
| **Precision** | 0.49 | 0.46 | 0.48 |
| **F1 score** | 0.40 | 0.39 | 0.39 |

**Supplementary Table S4. Performance of apneic event detection using LR.**

|  | **BCG features** | **BCG, BDR features** | **BCG, BDR, BDH features** |
| --- | --- | --- | --- |
| **Sensitivity** | 0.18 | 0.18 | 0.37 |
| **Precision** | 0.39 | 0.33 | 0.61 |
| **F1 score** | 0.25 | 0.23 | 0.46 |
